# VDR Activation Suppresses Pancreatic Cancer Metastasis Through Inhibition of the ERK Signaling Pathway

**DOI:** 10.3390/cancers18142296

**Published:** 2026-07-16

**Authors:** Wenjing Ding, Yanchun Fang, Hanmeng Xu, Xinyu Zhang, Chao Li, Yanping Wang, Daoxiang Zhang

**Affiliations:** 1 School of Basic Medical Sciences, Anhui Medical University, Hefei 230032, China; 2 Department of Pathology, Nantong Haimen District People’s Hospital, Nantong 226100, China; 3 School of Life Sciences, Anhui Medical University, Hefei 230032, China

**Keywords:** VDR, ERK, calcipotriol, pancreatic cancer

## Abstract

The vitamin D receptor (VDR) has been implicated in tumor progression, but its functional role in pancreatic ductal adenocarcinoma (PDAC) metastasis remains unclear. Here, using pharmacological modulation, gain- and loss-of-function approaches, transcriptomic profiling, and an experimental lung colonization model, we demonstrate that VDR acts as a suppressor of PDAC metastasis. Pharmacological activation of VDR by calcipotriol did not affect tumor cell proliferation but markedly inhibited the migratory and invasive capacities of multiple PDAC cell lines. In contrast, genetic deletion or pharmacological inhibition of VDR significantly enhanced metastatic phenotypes. To investigate the underlying mechanisms, we performed RNA sequencing on PDAC cells with differential VDR expression following calcipotriol treatment. Pathway enrichment analysis identified MAPK/ERK signaling as one of the most prominently altered pathways upon VDR activation. Functional studies further demonstrated that ERK inhibition abrogated the pro-metastatic effects induced by VDR loss or inhibition. In vivo lung colonization assays confirmed that VDR deficiency markedly promoted pulmonary metastatic colonization. Collectively, these findings identify VDR as a critical suppressor of PDAC cell metastatic colonization and reveal a previously unrecognized VDR–ERK regulatory axis that may represent a potential therapeutic target for limiting metastatic progression in pancreatic cancer.

## Simple Summary

Pancreatic cancer features aggressive metastasis and poor prognosis, and effective targeted strategies to block tumor spread are urgently needed. The protein vitamin D receptor is known to regulate tumor progression, yet its specific function and molecular mechanism in pancreatic cancer metastasis remain unclear. This study explored the biological effects and downstream pathway of this receptor in pancreatic cancer. We verified that activating this protein restrains cancer cell migration and lung metastasis, while its loss accelerates tumor spread by boosting core cell signaling. These findings clarify a new regulatory mechanism driving pancreatic cancer metastasis and offer a promising target for anti-metastatic therapy.

## 1. Introduction

Pancreatic ductal adenocarcinoma (PDAC) remains one of the most lethal malignancies, with a 5-year survival rate below 12% and an exceptionally high propensity for early metastasis [1,2]. Metastatic dissemination rather than primary tumor growth accounts for the majority of PDAC-related mortality [3,4,5], underscoring the need to identify signaling pathways that restrain tumor spread and may serve as therapeutic targets. Despite advances in genomic characterization and molecular classification, the regulatory networks governing PDAC metastasis are still incompletely understood.

The vitamin D receptor (VDR), a nuclear hormone receptor activated by vitamin D and analogs, such as calcipotriol (Cal), has attracted attention for its context-dependent roles in cancer biology [6,7,8]. Cal, a synthetic vitamin D analog with lower calcemic activity than calcitriol (1,25-dihydroxyvitamin D_3_; 1,25(OH)_2_D_3_), has been widely used experimentally and clinically because of its favorable safety profile [9,10,11]. While VDR signaling had affected cell adhesion and inhibited cell invasive capacity, with many of these effects being associated with a more differentiated phenotype [12,13,14] and stromal remodeling in several tumor types [15,16], its function in PDAC remains controversial. Previous studies have suggested that VDR activation in cancer-associated fibroblasts can reprogram the tumor stroma and improve therapeutic delivery [17,18], whereas the direct role of VDR signaling within PDAC epithelial cells themselves is far less defined. In particular, whether VDR influences metastatic behavior and the molecular mechanisms that mediate such effects have not been systematically addressed.

In this study, we uncover a tumor-cell-intrinsic role of VDR as a suppressor of PDAC metastasis. We show that pharmacological activation of VDR by Cal does not affect PDAC cell proliferation but profoundly inhibits migratory and invasive capacities. Conversely, VDR deletion or pharmacological inhibition enhances metastatic phenotypes in vitro and promotes lung colonization in vivo. Transcriptomic profiling of PDAC cells with differential VDR expression identifies mitogen-activated protein kinase/extracellular signal-regulated kinase (MAPK/ERK) signaling as a major pathway altered upon VDR activation. Functional validation further demonstrates that extracellular signal-regulated kinase (ERK) activity is required for VDR-mediated suppression of metastatic traits.

Together, these findings reveal an unexpected VDR–ERK regulatory axis that restrains PDAC metastasis and highlight VDR as a potential therapeutic vulnerability for limiting metastatic progression in pancreatic cancer.

## 2. Results

### 2.1. Activation of Vitamin D Receptor Suppresses Pancreatic Cancer Cell Migration Without Affecting Proliferation

**Figure 1:**
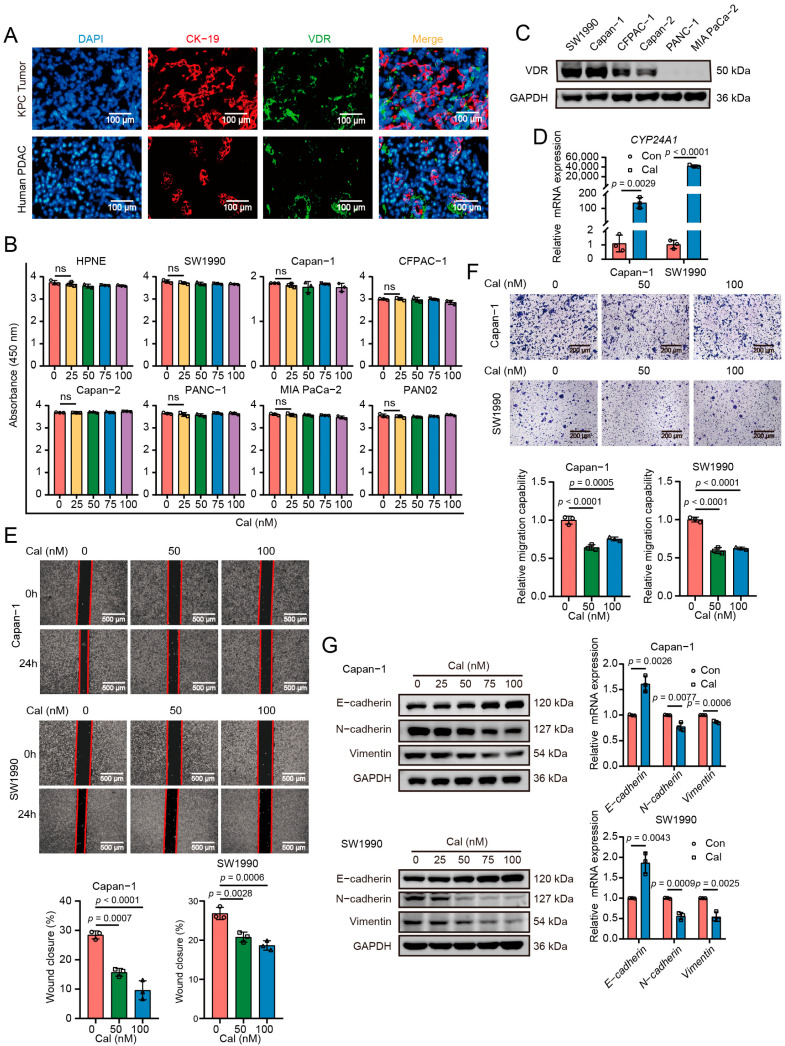
Activation of the vitamin D receptor suppresses pancreatic cancer cell migration without markedly affecting proliferation. (**A**) Representative immunofluorescence images showing VDR expression in pancreatic tumor tissues from KPC mice and human PDAC specimens. Nuclei were counterstained with DAPI. Scale bar, 100 μm. Representative images from three independent samples are shown. (**B**) Cell proliferation analysis of pancreatic cancer cell lines and HPNE cells treated with calcipotriol at the indicated concentrations of 0, 25, 50, 75, and 100 nM for 48 h. Cell viability/proliferation was measured using the CCK-8 assay. Data are presented as mean ± SD from three independent experiments. (**C**) Western blotting analysis of VDR protein expression in different pancreatic cancer cell lines. GAPDH was used as a loading control. The experiment was repeated independently three times with similar results. (**D**) QRT-PCR analysis showing the induction of *CYP24A1*, a canonical VDR target gene, in Capan-1 and SW1990 cells after calcipotriol treatment. GAPDH was used as the internal control. Data are presented as mean ± SD from three independent experiments. (**E**) Representative wound-healing images showing the migratory capacity of Capan-1 and SW1990 cells treated with calcipotriol at 50 nM for 24 h. Images were captured at 0 h and 24 h after scratching. The wound closure area was quantified using ImageJ software (ImageJ 1.53r). Scale bar, 500 μm. (**F**) Representative images and quantification of Transwell migration assays in Capan-1 and SW1990 cells treated with calcipotriol at 50 nM for 48 h. Migrated cells on the lower surface of the membrane were fixed, stained with 0.1% (*w*/*v*) crystal violet, and counted in three randomly selected fields per well. Scale bar, 200 μm. (**G**) Western blotting and qRT-PCR analysis of EMT markers, including E-cadherin, N-cadherin, and Vimentin, in Capan-1 and SW1990 cells treated with calcipotriol at 50 nM for 48 h. GAPDH was used as a loading control for Western blotting, and GAPDH was used as the internal control for qRT-PCR. Data are shown as mean ± SD from three independent experiments.

Vitamin D receptor (VDR) has been reported to play highly context-dependent roles in cancer, functioning either as a tumor suppressor or promoter depending on tumor type and microenvironment, and is involved in regulating proliferation, differentiation, inflammation, metabolism, and tumor microenvironment remodeling [19,20]. To investigate the role of VDR in pancreatic cancer, we first investigated its expression patterns in pancreatic tumor tissues of KPC mice (LSL-K-RasG12D; LSL-Trp53R172H; Pdx1-Cre) [21] and human PDAC tissues using immunofluorescence staining. We found that VDR expression was heterogeneous, with only a subset of tumor cells showing detectable levels, likely reflecting intratumoral heterogeneity (Figure 1A). To investigate the functional role of VDR activation, we treated a panel of pancreatic cancer cell lines (SW1990, Capan-1, CFPAC-1, Capan-2, PANC-1, MIA PaCa-2, PAN02) and an immortalized pancreatic ductal cell line (HPNE) with the VDR agonist Cal. Cell proliferation assays showed no significant change upon Cal treatment compared to controls (Figure 1B). We next detected endogenous VDR protein levels in six PDAC cell lines using Western blotting and selected two cell lines with high basal VDR expression (SW1990 and Capan-1) and one cell line with low VDR expression (PANC-1) for subsequent functional validation (Figure 1C). To confirm activation of the VDR signaling pathway, we examined the expression of the classical VDR target gene *CYP24A1* by qPCR and found that Cal treatment significantly induced *CYP24A1* expression (Figure 1D). Given the negligible effect of VDR activation on cell proliferation, we further investigated its potential role in regulating pancreatic cancer metastatic phenotypes. In wound healing assays (VDR-high-expressing Capan-1 and SW1990 cell lines), Cal treatment significantly reduced the migration of pancreatic cancer cell lines. Consistently, Transwell assays demonstrated a marked decrease in migration capacity after Cal exposure (Figure 1E,F). To further investigate whether VDR activation affects epithelial–mesenchymal transition (EMT), we examined the expression of EMT markers by Western blotting and quantitative PCR. Cal treatment induced upregulation of E-cadherin and downregulation of N-cadherin and Vimentin protein levels in Capan-1 and SW1990 cells. These changes were mirrored at the mRNA level (Figure 1G), indicating that VDR activation suppresses EMT in pancreatic cancer cells.

### 2.2. VDR Knockout or Pharmacological Inhibition Enhances Migration in Pancreatic Cancer Cells

**Figure 2:**
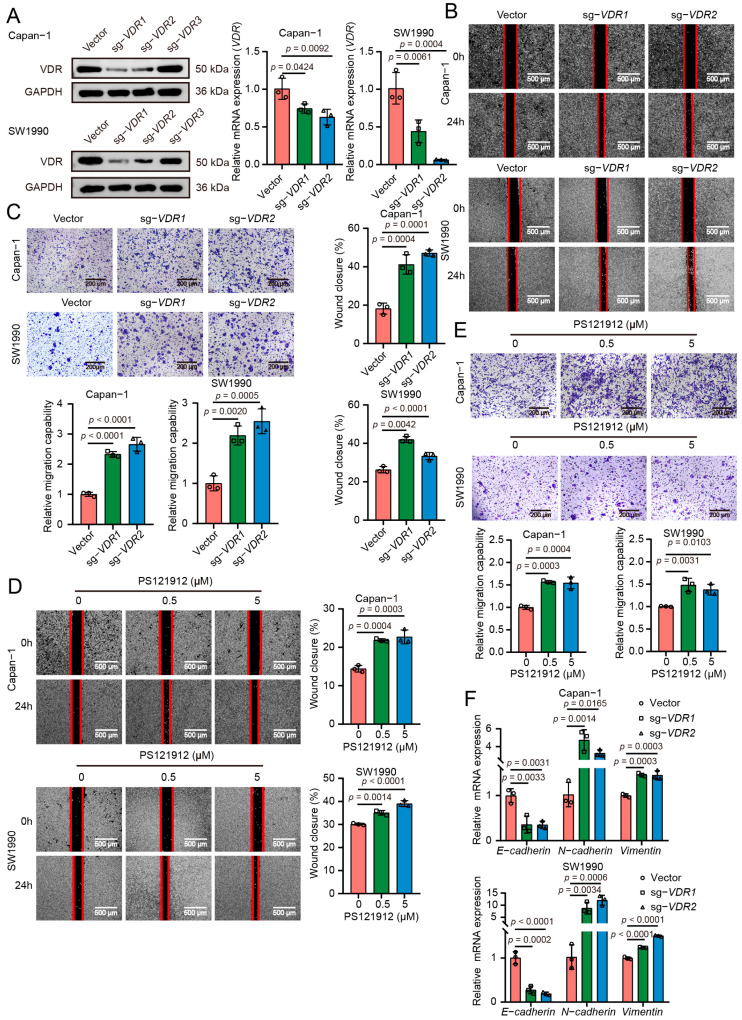
VDR knockout or pharmacological inhibition enhances migration in pancreatic cancer cells. (**A**) Validation of VDR knockout in Capan-1 and SW1990 cells generated using CRISPR/Cas9 technology. VDR expression was examined by Western blotting and qRT-PCR. GAPDH was used as a loading control, and GAPDH was used as the internal control for qRT-PCR. (**B**) Representative wound-healing images and quantification showing increased migratory ability of VDR-knockout Capan-1 and SW1990 cells compared with control cells. Images were acquired at 0 h and 24 h after scratching. Scale bar, 500 μm. (**C**) Representative images and quantification of Transwell migration assays showing enhanced migration of VDR-knockout Capan-1 and SW1990 cells. Migrated cells were stained with 0.1% (*w*/*v*) crystal violet and counted in three randomly selected microscopic fields per well. Scale bar, 200 μm. (**D**) Representative wound-healing images and quantification showing increased migration of Capan-1 and SW1990 cells treated with the VDR inhibitor PS121912 at 0, 0.5, and 5 nM for 24 h. Images were acquired at 0 h and 24 h after scratching. Scale bar, 500 μm. (**E**) Representative images and quantification of Transwell migration assays showing enhanced migration of Capan-1 and SW1990 cells following treatment with PS121912 at 0, 0.5, and 5 μM for 48 h. Migrated cells were quantified from three randomly selected fields per well. Scale bar, 200 μm. (**F**) QRT-PCR analysis of EMT markers, including E-cadherin, N-cadherin, and Vimentin, in control and VDR-knockout Capan-1 and SW1990 cells. GAPDH was used as the internal control for qRT-PCR.

To further validate the role of VDR in EMT, we employed CRISPR/Cas9 technology to knockout VDR in Capan-1 and SW1990 cells. Successful VDR depletion by two independent sgRNAs (sg-VDR1 and sg-VDR2) was confirmed by quantitative PCR and Western blotting (Figure 2A). To further validate the role of VDR in pancreatic cancer cell migration and EMT, we additionally established VDR-overexpressing and rescue cell lines in Capan-1 and SW1990 cells, and the transfection efficiency was confirmed by Western blotting (Supplementary Figure S1A). Functional assays revealed that VDR knockout significantly enhanced the migratory capacity of pancreatic cancer cells in wound healing assays. Consistently, Transwell assays demonstrated increased migration potential following VDR deletion (Figure 2B,C). Conversely, VDR overexpression inhibited cell migration, while VDR rescue attenuated the increased migratory capacity induced by VDR knockout (Supplementary Figure S1B,C). To corroborate these findings, we treated pancreatic cancer cells with the VDR inhibitor PS121912 [22,23]. Similar to genetic knockout, pharmacological inhibition of VDR promoted cell migration in both wound healing and Transwell assays (Figure 2D,E). At the molecular level, VDR knockout resulted in decreased expression of the epithelial marker E-cadherin, concomitant with upregulation of mesenchymal markers N-cadherin and Vimentin at mRNA levels (Figure 2F). In contrast, VDR overexpression reversed these EMT-related changes, whereas re-expression of VDR in VDR-knockout cells attenuated the EMT phenotype induced by VDR depletion (Supplementary Figure S1D). Collectively, these results demonstrate that VDR suppresses EMT and mitigates metastasis-associated malignant phenotypes in pancreatic cancer cells.

### 2.3. VDR Regulates ERK/MAPK Signaling in Pancreatic Cancer Cells

**Figure 3:**
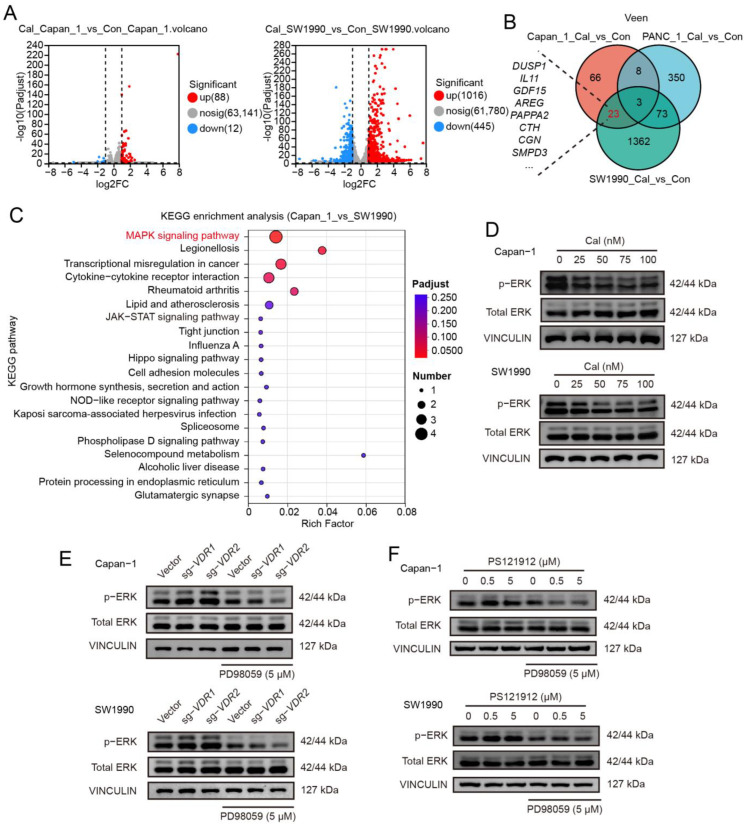
VDR regulates ERK/MAPK signaling in pancreatic cancer cells. (**A**) Volcano plot showing differentially expressed genes in Capan-1 and SW1990 cells following calcipotriol treatment at 50 nM for 48 h, based on RNA sequencing analysis. Differentially expressed genes were defined as |log_2_ fold change| ≥ 1 and adjusted *p* value < 0.05. (**B**) Venn diagram showing overlapping differentially expressed genes shared by high-VDR-expressing Capan-1 and SW1990 cells after calcipotriol treatment but not observed in low-VDR-expressing PANC-1 cells. (**C**) KEGG pathway enrichment analysis of overlapping differentially expressed genes altered by calcipotriol treatment. The enriched pathways were ranked according to the enrichment score. Significantly enriched pathways were defined as adjusted *p* value < 0.05. (**D**) Western blotting analysis of phosphorylated ERK and total ERK levels in Capan-1 and SW1990 cells treated with calcipotriol at 0, 25, 50, 75, and 100 nM for 48 h. Total ERK and VINCULIN were used as loading controls. (**E**) Western blotting analysis showing increased p-ERK levels in VDR-knockout Capan-1 and SW1990 cells and their reduction after treatment with the ERK inhibitor PD98059 at 5 µM for 48 h. Total ERK and VINCULIN were used as controls. (**F**) Western blotting analysis showing increased p-ERK levels in Capan-1 and SW1990 cells treated with the VDR inhibitor PS121912 at 0, 0.5, and 5 µM for 48 h, with reversal by PD98059 treatment at 5 µM for 48 h. Total ERK and VINCULIN were used as controls.

To dissect the downstream molecular mechanisms underlying VDR-mediated regulation of PDAC metastasis, we treated cells with the VDR agonist Cal and performed RNA sequencing to profile transcriptomic alterations based on the differential VDR expression among various cell lines presented in Figure 1C. RNA-seq analysis revealed substantial heterogeneity between cell lines: in Capan-1 cells, 88 genes were upregulated and 12 downregulated upon Cal treatment, whereas in SW1990 cells, 1016 genes were upregulated and 445 downregulated (Figure 3A). Venn diagram analysis identified 23 genes whose expression changed in both SW1990 and Capan-1 cells but not in PANC-1 cells, including genes previously implicated in EMT, such as *DUSP1*, *IL11*, *GDF15*, and *AREG*. KEGG pathway enrichment analysis demonstrated significant alterations in the MAPK signaling pathway following Cal treatment (Figure 3B,C). To validate these findings, we assessed ERK phosphorylation (p-ERK) levels by Western blotting. Activation of VDR by Cal reduced p-ERK levels (Figure 3D), while VDR knockout markedly increased p-ERK. In contrast, VDR overexpression significantly reduced ERK phosphorylation levels, whereas rescue treatment did not obviously alter p-ERK expression (Supplementary Figure S1E). Treatment with the ERK inhibitor PD98059 reversed the increase in p-ERK caused by VDR deletion (Figure 3E). Conversely, the VDR inhibitor PS121912 induced p-ERK expression, which was also suppressed by PD98059 (Figure 3F). Collectively, these data indicate that VDR modulates the ERK/MAPK signaling pathway in pancreatic cancer cells.

### 2.4. VDR Regulates EMT in Pancreatic Cancer via the ERK/MAPK Signaling Pathway

**Figure 4:**
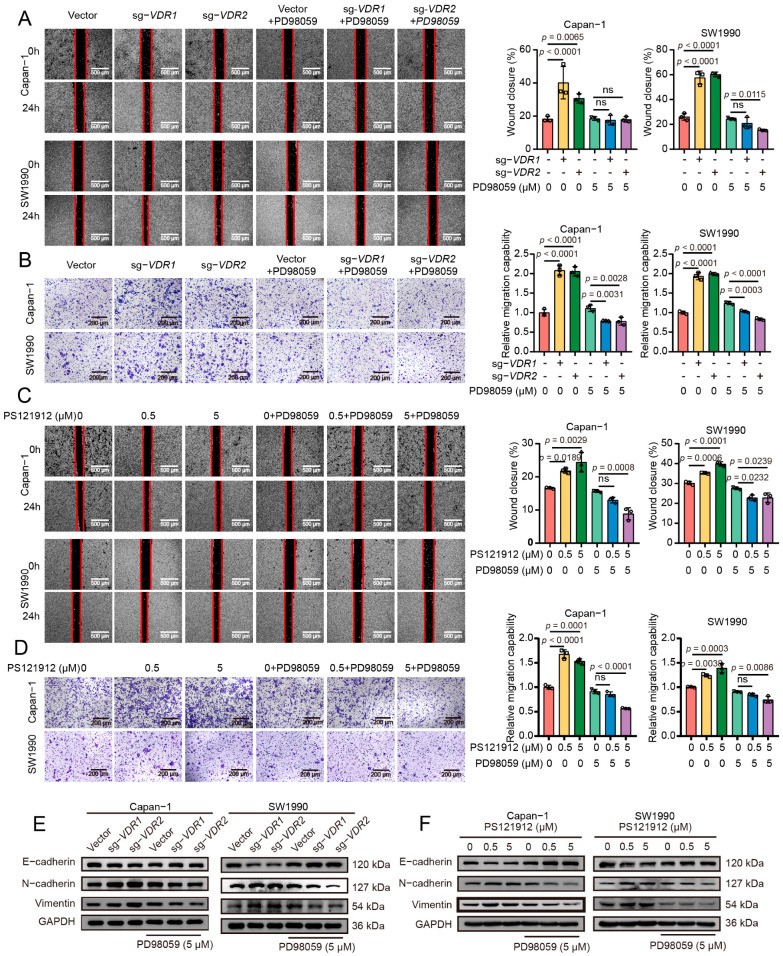
VDR regulates EMT in pancreatic cancer through the ERK/MAPK signaling pathway. (**A**) Representative wound-healing images and quantification showing that PD98059 treatment suppresses the enhanced migration induced by VDR knockout in Capan-1 and SW1990 cells. Cells were treated with PD98059 at 5 µM for 24 h. Images were captured at 0 h and 24 h after scratching. Scale bar, 500 μm. (**B**) Representative images and quantification of Transwell migration assays showing that PD98059 inhibits the increased migratory ability caused by VDR knockout. Migrated cells were stained with 0.1% (*w*/*v*) crystal violet and counted in three randomly selected fields per well. Scale bar, 200 μm. (**C**) Representative wound-healing images and quantification showing that PD98059 blocks the enhanced migration induced by pharmacological inhibition of VDR with PS121912. Cells were treated with PS121912 at 0, 0.5, and 5 µM and/or PD98059 at 5 µM for 24 h. Scale bar, 500 μm. (**D**) Representative images and quantification of Transwell migration assays showing that PD98059 suppresses the increased migration resulting from VDR inhibition. Migrated cells were counted in three randomly selected microscopic fields per well. Scale bar, 200 μm. (**E**) Western blotting analysis showing that PD98059 reverses EMT marker changes caused by VDR knockout, as indicated by increased E-cadherin and decreased N-cadherin and Vimentin expression. GAPDH was used as the internal control for qRT-PCR. (**F**) Western blotting analysis showing that PD98059 similarly reverses EMT marker alterations induced by PS121912-mediated VDR inhibition in Capan-1 and SW1990 cells. GAPDH was used as the internal control for qRT-PCR.

To investigate whether VDR regulates EMT through the ERK/MAPK pathway in pancreatic cancer, we utilized VDR knockout cells. In wound healing assays, treatment with the ERK inhibitor PD98059 significantly suppressed the enhanced migration induced by VDR deletion. Similarly, Transwell migration assays demonstrated that PD98059 effectively inhibited the increased migratory capacity resulting from VDR knockout (Figure 4A,B). At the pharmacological level, ERK inhibition also reversed the pro-migratory effects induced by the VDR inhibitor. Specifically, in wound healing and Transwell assays, PD98059 markedly attenuated the migration promoted by VDR inhibition (Figure 4C,D). We further assessed EMT marker expression and found that PD98059 restored E-cadherin levels reduced by VDR knockout while decreasing the elevated expression of N-cadherin and Vimentin. Consistent results were observed when ERK inhibition was applied following pharmacological suppression of VDR (Figure 4E,F). Collectively, these findings indicate that VDR modulates EMT in pancreatic cancer cells by regulating the MAPK/ERK signaling pathway.

### 2.5. VDR Deletion Enhances Lung Metastasis of Pancreatic Cancer In Vivo

**Figure 5:**
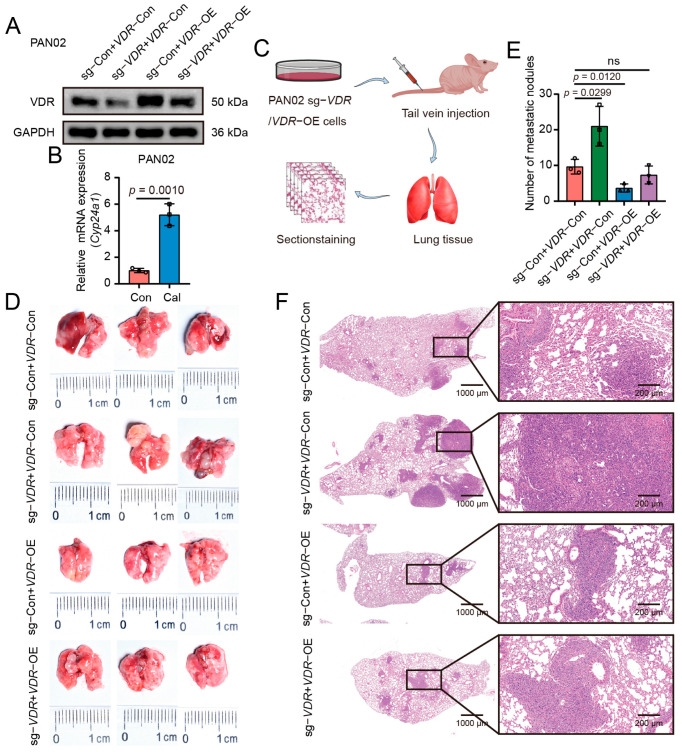

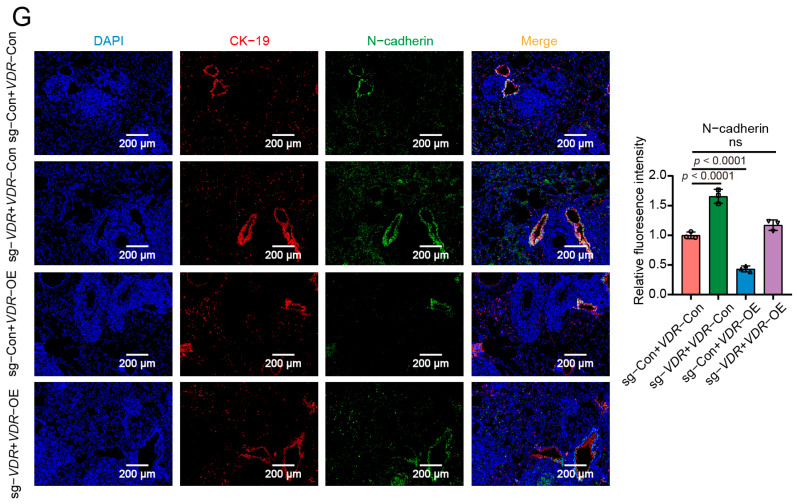
VDR inhibits lung metastasis of pancreatic cancer cells in vivo. (**A**) VDR knockout, overexpression, and rescue cell lines were established in PAN02 cells via the CRISPR/Cas9 system and lentiviral overexpression plasmids, and the transfection efficiency was verified. VDR protein expression was detected by Western blotting, with GAPDH serving as the loading control. (**B**) QRT-PCR analysis showing the induction of *Cyp24a1*, a canonical VDR target gene, in PAN02 pancreatic cancer cells after calcipotriol treatment. GAPDH was used as the internal control. Data are presented as mean ± SD from three independent experiments. (**C**) Schematic diagram of the experimental lung metastasis model. Four groups of pancreatic cancer cells, including the control group, VDR-knockout group, VDR-overexpressing group, and VDR rescue group, were injected into 6-week-old female BALB/c nude mice via the tail vein at a dose of 2 × 10^6^ cells per mouse. Mice were sacrificed 8 weeks post-injection, and lung tissues were harvested to analyze metastatic lesions. (**D**) Representative gross images of lung metastases and quantification of metastatic nodules in mice injected with the control group, VDR-knockout group, VDR-overexpressing group, and VDR rescue group pancreatic cancer cells. Metastatic nodules on the lung surface were counted manually in a blinded manner. n = 3 mice per group. (**E**) Quantitative analysis histogram of lung metastatic nodules in mice injected with pancreatic cancer cells. (**F**) Representative hematoxylin and eosin staining images showing metastatic lesions in lung tissues from mice injected with the control group, VDR-knockout group, VDR-overexpressing group and VDR rescue group pancreatic cancer cells. Scale bar, 1000/200 μm. (**G**) Representative immunofluorescence staining images and quantitative analysis show that VDR inhibits N-cadherin expression in lung metastatic lesions derived from pancreatic cancer cells. Nuclei were counterstained with DAPI, and fluorescence intensity was quantified using ImageJ software. Scale bar, 200 μm.

To investigate the in vivo role of VDR in regulating pancreatic cancer metastasis colonization, we constructed a tail vein injection lung metastasis model with VDR-knockout (sg-VDR), VDR-overexpressing (VDR-OE), and VDR-rescue PAN02 cell lines, whose transfection efficiency was verified via Western blotting (Figure 5A). To verify the functional activation of VDR signaling in vivo, we detected the mRNA level of the canonical VDR target gene *Cyp24a1* by qPCR, and the results confirmed that Cal treatment significantly induced *Cyp24a1* expression (Figure 5B). Following intravenous cell injection, all mice were tracked for lung metastatic lesion formation (Figure 5C). Macroscopic lung tissue observation showed more and larger metastatic lesions in the sg-VDR group relative to controls; VDR overexpression lessened lesion burden, and VDR rescue reversed the aggressive metastatic phenotype caused by VDR knockout (Figure 5D,E). Hematoxylin and eosin (HE) staining further validated this trend: the VDR-deficient group displayed severe cancer cell infiltration and damaged lung structure, while infiltration was alleviated in the VDR-overexpressing group and restored to normal levels upon VDR rescue (Figure 5F). To uncover molecular mechanisms underlying metastasis promotion, we conducted immunofluorescence staining of lung tissues to detect core EMT markers. Consistently, the fluorescence intensity of the mesenchymal marker N-cadherin was significantly elevated in metastatic lesions derived from sg-VDR cells. As expected, VDR overexpression downregulated N-cadherin expression, and VDR rescue effectively abolished the upregulation of N-cadherin induced by VDR ablation (Figure 5G). Such EMT marker shifts in lung metastases suggest that VDR loss induces in vivo EMT to boost pancreatic cancer cell spread and lung colonization. Collectively, these in vivo experimental results demonstrate that VDR exerts a critical tumor-suppressive function during pancreatic cancer progression.

## 3. Discussion

Our study demonstrates that activation of the vitamin D receptor (VDR) suppresses pancreatic cancer cell migration, invasion, and EMT-associated phenotypes without markedly affecting cell proliferation. We observed heterogeneous VDR expression in pancreatic tumor tissues, suggesting that VDR signaling may vary among tumor cell populations and different compartments of the tumor microenvironment. Using in vitro assays, transcriptomic analysis, VDR gain- and loss-of-function models, and in vivo experiments, we identified a tumor-cell-intrinsic role of VDR in restraining metastatic traits in PDAC.

Previous studies have shown that VDR exerts context-dependent functions in cancer [19,24]. In PDAC, VDR signaling has mainly been linked to stromal remodeling and chemotherapy response [18,25], whereas its direct role in tumor cell migration and EMT remains less clear. Our findings extend these studies by showing that VDR activation directly suppresses metastatic phenotypes in pancreatic cancer cells.

Vitamin D signaling is complex and not limited to the classical vitamin D–VDR axis. CYP11A1-mediated metabolism can generate biologically active vitamin D and lumisterol metabolites, which may act through VDR as well as alternative receptors, including RORα/RORγ, LXRα/LXRβ, and AhR [26,27,28]. Therefore, although our study focuses on calcipotriol-induced VDR signaling, possible contributions from non-canonical vitamin D pathways or VDR-independent mechanisms cannot be fully excluded.

RNA-seq analysis showed that VDR activation altered genes related to EMT and MAPK signaling, including DUSP1, IL11, GDF15, and AREG. These findings suggest that VDR may regulate a broader transcriptional network involved in cellular plasticity and metastatic potential. However, the functional roles of these genes in VDR-mediated EMT suppression require further validation.

Mechanistically, we found that VDR activation reduced ERK phosphorylation, whereas VDR knockout or pharmacological inhibition increased ERK activation, EMT marker changes, and migratory and invasive abilities. Importantly, ERK inhibition with PD98059 reversed these effects, indicating that VDR suppresses EMT-associated metastatic phenotypes, at least in part, through inhibition of ERK/MAPK signaling.

In vivo, VDR deficiency increased lung metastatic burden in the tail vein injection model and was accompanied by decreased E-cadherin and increased N-cadherin expression in metastatic lesions. These results support a role for VDR in limiting metastatic colonization. Therapeutically, our findings suggest that VDR agonists may help suppress PDAC metastasis, and their combination with ERK/MAPK pathway inhibitors may be a potential strategy for tumors with retained VDR expression and active MAPK signaling. However, this possibility requires further validation in clinically relevant models.

Several limitations should be acknowledged. First, VDR expression is heterogeneous in PDAC, which may influence the response to VDR-targeted treatment. Second, although calcipotriol is widely used as a VDR agonist, potential off-target or VDR-independent effects cannot be excluded. Third, our study mainly focused on ERK/MAPK signaling, while other PDAC-related pathways, such as PI3K/AKT, TGF-β, Wnt/β-catenin, NF-κB, and stromal or immune-related pathways, may also be involved. Fourth, because the in vivo experiments were performed using a tail-vein injection model, our findings primarily reflect the role of VDR in lung metastatic colonization rather than the entire spontaneous metastatic process originating from primary pancreatic tumors. Future studies using orthotopic or genetically engineered PDAC models will be important to further validate these findings [29,30]. Finally, ChIP-seq, VDR reporter assays, and orthotopic or patient-derived models are needed to define direct VDR targets and evaluate translational relevance.

In conclusion, our study identifies a tumor-cell-intrinsic role of VDR in suppressing PDAC migration, invasion, and EMT-associated metastatic traits. These findings reveal a VDR–ERK/MAPK regulatory axis and provide a rationale for further investigation of VDR-based therapeutic strategies to limit PDAC metastasis.

## 4. Materials and Methods

### 4.1. Cell Culture and Reagents

The human pancreatic cancer cell lines Capan-1, SW1990, CFPAC-1, Capan-2, PANC-1, MIA PaCa-2, PAN02, and the immortalized pancreatic ductal epithelial cell line HPNE were purchased from the BeNa Culture Collection (Beijing, China). All cell lines were cultured in high-glucose DMEM supplemented with 10% fetal bovine serum (FBS) and 1% penicillin/streptomycin at 37 °C in a humidified incubator containing 5% CO_2_. VDR-knockout Capan-1 and PAN02 cells were generated using the LentiCRISPR v2 system. The VDR-targeting plasmid and empty backbone control plasmid (LentiCRISPR v2-Puro) were purchased from Tsingke Biotechnology Co., Ltd. (Beijing, China). Stable cells were selected using puromycin, and VDR knockout efficiency was validated by Western blotting. The VDR agonist Cal, MEK inhibitor PD98059, and VDR inhibitor PS121912 were purchased from MedChemExpress (MCE, Shanghai, China). The final concentration of DMSO in all treatments was maintained below 0.1%.

### 4.2. Cell Proliferation Assay

To evaluate the effects of different treatments on pancreatic cancer cell viability, cells were seeded in 96-well plates at a density of 4 × 10^3^ cells per well and allowed to adhere for 24 h. The cells were then exposed to the indicated compounds for 48 h, with equal volumes of DMSO used as a vehicle control. Cell viability was assessed using the Cell Counting Kit-8 (CCK-8; Taoshu, Shanghai, China). Briefly, 10 μL of CCK-8 solution was added to each well and incubated at 37 °C for 4 h. Absorbance at 450 nm was measured using a Spark multimode microplate reader (Tecan, Männedorf, Switzerland).

### 4.3. Cell Wound Healing Assay

Cells were seeded in 6-well plates at a density of 2 × 10^6^ cells per well and allowed to adhere for 24 h. A sterile 200-μL pipette tip was then used to create a linear scratch across the cell monolayer along the pre-marked guidelines. Wells were gently washed with PBS to remove debris and subsequently cultured in serum-free medium containing the indicated treatments for 24 h. Images of the wound area were captured at 0 h and 24 h using an inverted microscope (Mshot, Guangzhou, China). Wound closure was quantified in ImageJ, and the relative migration rate (%) was calculated as [(wound area at 0 h − wound area at 24 h)/wound area at 0 h] × 100%. All experiments were performed in triplicate.

### 4.4. Transwell Migration Assay

Capan-1 and SW1990 pancreatic cancer cells (6 × 10^4^ and 4 × 10^5^ cells, respectively) were resuspended in 300 μL of serum-free DMEM and seeded into the upper chambers of 24-well Transwell inserts (LABSELECT, Beijing, China). The lower chambers were filled with 600 μL of DMEM supplemented with 10% fetal bovine serum as a chemoattractant. Cells were incubated at 37 °C with 5% CO_2_ for 48 h. After incubation, the medium was removed, and cells were washed with PBS and fixed with 4% paraformaldehyde (Biosharp, Beijing, China) for 15 min at room temperature. Following PBS washes, cells were stained with 0.1% crystal violet for 15 min. Non-migrated cells on the upper surface of the membrane were carefully removed with a cotton swab. Images of migrated cells on the lower membrane surface were acquired using an inverted microscope, and quantification was performed using ImageJ software.

### 4.5. Protein Extraction and Western Blotting

Cells were digested with an appropriate amount of trypsin and pelleted by centrifugation at 12,000 rpm for 5 min at 4 °C. Pancreatic cancer cells were lysed in RIPA buffer (Biosharp, Beijing, China) supplemented with 10% protease inhibitor cocktail. Protein concentrations were determined using a BCA assay kit (Beyotime, Shanghai, China) according to the manufacturer’s instructions. Equal amounts of protein were separated on 8–12% SDS–polyacrylamide gels and transferred onto polyvinylidene fluoride (PVDF) membranes. Membranes were blocked with 5% bovine serum albumin in TBST for 1 h at room temperature and then incubated with the appropriate primary antibodies overnight at 4 °C. After washing with TBST, membranes were incubated with species-matched HRP-conjugated secondary antibodies for 2 h. Protein signals were detected using Tanon ECL chemiluminescent substrate (Tanon, Shanghai, China) and imaged under dark conditions.

### 4.6. Real-Time PCR

Total RNA was isolated from pancreatic cancer cells using TRIzol reagent (GenStar, Beijing, China) according to the manufacturer’s protocol. One microgram of RNA was reverse-transcribed using random primers. Quantitative PCR was performed with SYBR Green I chemistry using a two-step cycling protocol: 95 °C for 30 s, followed by 40 cycles of 95 °C for 10 s and 60 °C for 30 s. Melting-curve analysis was conducted on a Roche LightCycler 96 system. Gene expression levels were normalized to GAPDH, and relative expression was calculated using the 2^−ΔΔCT^ method. All experiments were performed in triplicate.

### 4.7. Immunofluorescence Staining

Frozen tissue sections were equilibrated to room temperature for 10 min and fixed with 4% paraformaldehyde (Biosharp, China) for 15 min. After three washes with PBS, the sections were permeabilized with 0.1% Triton X-100 (Beyotime, China) for 15 min and subsequently blocked with 5% BSA (Biosharp, China) for 1 h at room temperature. The sections were then incubated overnight at 4 °C with primary antibodies diluted according to the manufacturer’s instructions. Following PBS washes, fluorescently labeled secondary antibodies were applied for 1 h at room temperature in the dark. After a final wash, the sections were mounted with anti-fade medium containing DAPI and allowed to sit for 10 min. Fluorescence images were acquired using an inverted fluorescence microscope (Aomei, Beijing, China), and relative signal intensity was quantified using ImageJ.

### 4.8. Tail-Vein Metastasis Model in BALB/c-Nude Mice

Six-week-old female BALB/c-Nude mice were purchased from GemPharmatech Co., Ltd. (Nanjing, Jiangsu, China). Mice were randomly assigned to experimental groups (n = 3 per group) using a random number table. Investigators were blinded to group allocation during animal handling, sample collection, and data analysis. All animal experiments were reviewed and approved by the Institutional Animal Care and Use Committee (IACUC) of Anhui Medical University (approval no. LLSC20261737) and were performed in accordance with the institutional guidelines for the care and use of laboratory animals. For the establishment of the experimental lung metastasis model, PAN02 pancreatic cancer cells of the control group, VDR knockout group, VDR overexpression group, and VDR knockout plus rescue overexpression group were intravenously injected into mice via the tail vein (2 × 10^6^ cells in 200 μL PBS per mouse). Mice were sacrificed 8 weeks after injection, and lung tissues were collected for subsequent histological and molecular analyses.

### 4.9. Transcriptome Sequencing

Transcriptome sequencing was performed by Majorbio Bio-pharm Technology (Shanghai, China). Three independent biological replicates were included for each group. RNA-seq libraries were prepared using the Illumina NovaSeq Reagent Kit according to the manufacturer’s instructions and sequenced on the Illumina NovaSeq 6000 platform. Each sample generated more than 6 Gb of clean data. Clean reads were aligned to the human reference genome (GRCh38) using HISAT2. Differential gene expression analysis was performed using DESeq2, and genes with a false discovery rate (FDR) < 0.05 and |log_2_ fold change| ≥ 1 were considered significantly differentially expressed. KEGG pathway enrichment analysis was conducted using KOBAS software (kobas-2.1.1). The raw sequence data reported in this study have been deposited in the Genome Sequence Archive (GSA-Human) at the National Genomics Data Center, China National Center for Bioinformation / Beijing Institute of Genomics, Chinese Academy of Sciences, under accession number HRA018378, and are publicly accessible at the NGDC GSA-Human database.

### 4.10. Statistical Analysis

Unless otherwise indicated, data are presented as mean ± standard deviation. Statistical differences between groups were assessed using either unpaired *t*-tests or one-way ANOVA, with *p* < 0.05 considered statistically significant. All experiments were performed at least three independent times.

## 5. Conclusions

In conclusion, this study solidly demonstrates that VDR serves as a vital metastasis suppressor in pancreatic cancer. By negatively modulating the core cell signaling pathway, this receptor inhibits malignant biological behaviors of cancer cells, including migration and EMT, and ultimately restrains tumor lung metastatic colonization. The newly identified regulatory linkage between VDR and cell signaling fills a research gap in pancreatic cancer metastatic mechanisms. It also provides a novel and reliable theoretical basis for developing targeted drugs to inhibit pancreatic cancer metastasis and improve patient prognosis.

## Data Availability

The data presented in this study are available in the article.

## Supplementary Materials

The following supporting information can be downloaded at https://www.mdpi.com/article/10.3390/cancers18142296/s1, Figure S1: (**A**) VDR knockout, overexpression and rescue cell lines were established in Capan-1 and SW1990 cells via the CRISPR/Cas9 system and lentiviral overexpression plasmids, and the transfection efficiency was verified. VDR protein expression was detected by Western blotting, with GAPDH serving as the loading control. (**B**) Representative images and quantitative analysis of wound healing assays. Compared with control cells, cell migratory capacity was markedly increased in VDR-knockout cells, obviously decreased in VDR-overexpressed cells, and showed no significant alteration in rescue cells. Images were captured at 0 h and 24 h after scratching. Scale bar, 500 μm. (**C**) Representative images and quantitative results of Transwell migration assays. VDR knockout significantly promoted cell migration, VDR overexpression inhibited migration, and rescue treatment exerted no obvious effect on the migratory ability of Capan-1 and SW1990 cells. Migrated cells were stained with 0.1% (*w*/*v*) crystal violet, and cell numbers were counted in three randomly selected fields per well. Scale bar, 200 μm. (**D**) Western blotting analysis of EMT-related markers, including E-cadherin, N-cadherin and Vimentin in control, VDR-knockout, VDR-overexpressed and rescue pancreatic cancer cells. GAPDH was used as the loading control. (**E**) Western blotting was performed to detect phosphorylated ERK levels in VDR-knockout, overexpressed and rescue Capan-1 and SW1990 cells. Total ERK and VINCULIN were used as internal controls.
