# Supplementary material for: VDR Activation Suppresses Pancreatic Cancer Metastasis Through Inhibition of the ERK Signaling Pathway
Source: Cancers (Basel). 2026 Jul 16;18(14):2296. doi: 10.3390/cancers18142296 (PMC13407328; doi:10.3390/cancers18142296)
Supplement: Supplementary file 1 [file cancers-18-02296-s001.zip › cancers-4423005-supplementary.pdf]

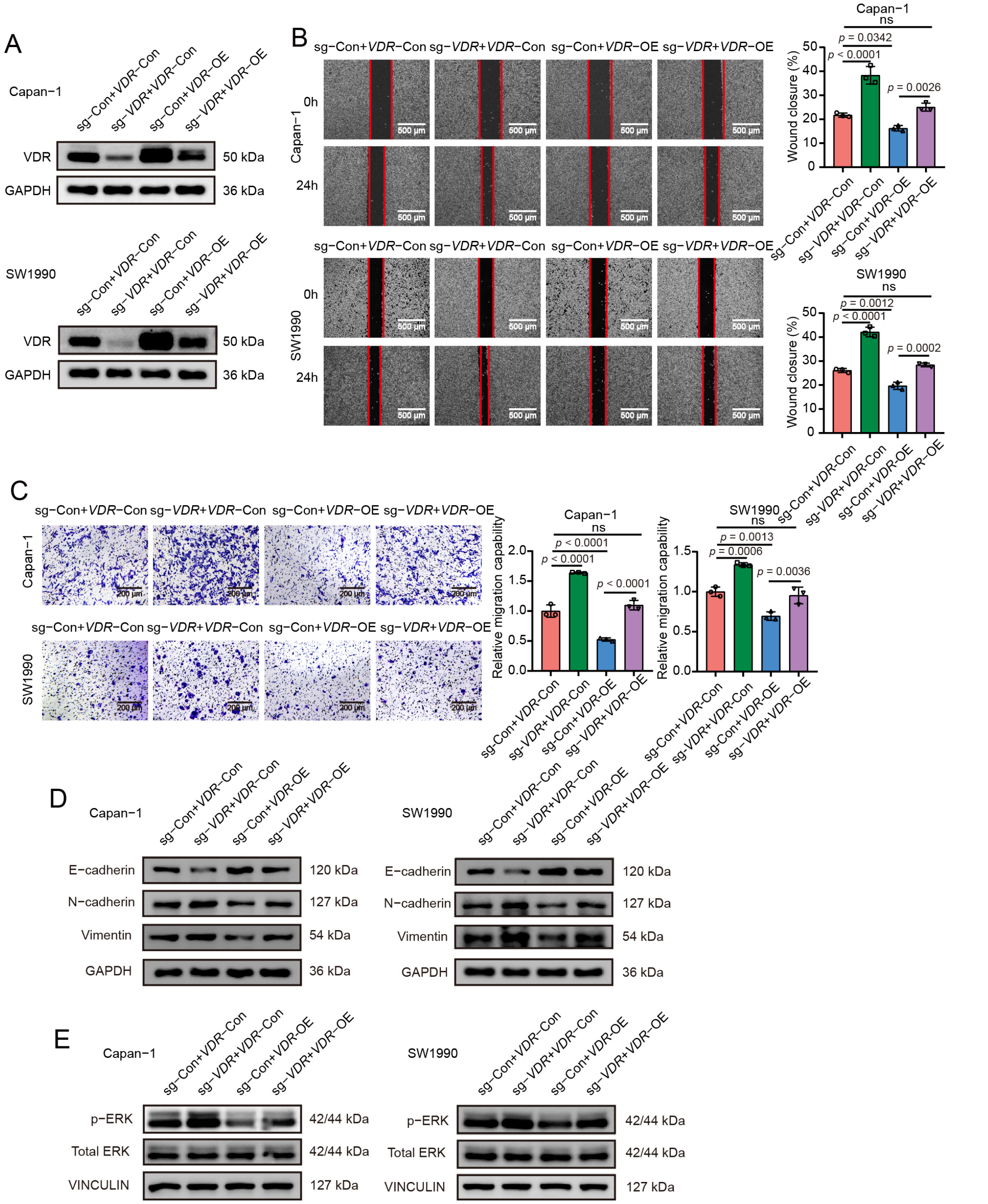

Figure S1: (**A**) VDR knockout, overexpression and rescue cell lines were established in Capan-1 and SW1990 cells via the CRISPR/Cas9 system and lentiviral overexpression plasmids, and the transfection efficiency was verified. VDR protein expression was detected by Western blotting, with GAPDH serving as the loading control. (**B**) Representative images and quantitative analysis of wound healing assays. Compared with control cells, cell migratory capacity was markedly increased in VDR-knockout cells, obviously decreased in VDR-overexpressed cells, and showed no significant alteration in rescue cells. Images were captured at 0 h and 24 h after scratching. Scale bar, 500 μm. (**C**) Representative images and quantitative results of Transwell migration assays. VDR knockout significantly promoted cell migration, VDR overexpression inhibited migration, and rescue treatment exerted no obvious effect on the migratory ability of Capan-1 and SW1990 cells. Migrated cells were stained with 0.1% (*w*/*v*) crystal violet, and cell numbers were counted in three randomly selected fields per well. Scale bar, 200 μm. (**D**) Western blotting analysis of EMT-related markers, including E-cadherin, N-cadherin and Vimentin in control, VDR-knockout, VDR-overexpressed and rescue pancreatic cancer cells. GAPDH was used as the loading control. (**E**) Western blotting was performed to detect phosphorylated ERK levels in VDR-knockout, overexpressed and rescue Capan-1 and SW1990 cells. Total ERK and VINCULIN were used as internal controls.
